# Acknowledgments

**Published:** 2004

## OUTSIDE ADVISORS

Adrian Angold, M.R.C. Psych
  Duke University
  Psychiatry and Behavioral Sciences
  Box 3454, Brightleaf Square, Suite 22
  Durham, NC 22710–3454
Professor Richard Bonnie
  John S. Battle Professor of Law
  Director, University of Virginia Institute of Law, Psychiatry and Public Policy
  University of Virginia School of Law
  P.O. Box 400405
  Charlottesville, VA 22904–4405
Jane Brown, Ph.D.
  James L. Knight Professor
  Journalism/Mass Communications
  360 Carroll Hall
  University of North Carolina
  Chapel Hill, NC 27514
Sandra Brown, Ph.D.
  Professor of Psychology and Psychiatry
  Department of Psychology, 0109
  University of California, San Diego
  9500 Gilman Drive
  La Jolla, CA 92093–0109
Ronald E. Dahl, M.D.
  Staunton Professor of Psychiatry and Pediatrics
  Western Psychiatric Institute and Clinic
  University of Pittsburgh
  3811 O’Hara Street
  Pittsburgh, PA 15213
Thomas J. Dishion, Ph.D.
  Founder and Director of Research
  Child and Family Center
  University of Oregon
  195 North 12th Avenue
  Eugene, OR 97401
John E. Donovan, Ph.D.
  Department of Psychiatry
  Western Psychiatric Institute and Clinic
  University of Pittsburgh School of Medicine
  3811 O’Hara Street
  Pittsburgh, PA 15213
Cindy L. Ehlers, Ph.D.
  Department of Neuropharmacology
  The Scripps Research Institute
  10550 N. Torrey Pines Road
  CVN–14
  La Jolla, CA 92037
Mrs. Kendel Ehrlich
  First Lady of Maryland
  Office of the First Lady
  State House
  100 State Circle
  Annapolis, MD 21401–1925
Ms. Mimi Fleury
  Community of Concern
  c/o Georgetown Preparatory School
  10900 Rockville Pike
  North Bethesda, MD 20852
Mrs. Nancy Freudenthal
  First Lady of Wyoming
  Office of the First Lady
  5001 Central Avenue
  Cheyenne, WY 82009–4011
Jay Giedd, M.D.
  National Institutes of Health
  National Institute of Mental Health
  9000 Rockville Pike, MSC 1367
  Bethesda, MD 20892–1367
Jennifer Lerner, Ph.D.
  Department of Social and Decision Sciences
  Carnegie Mellon University
  208 Porter Hall
  Pittsburgh, PA 15213
Christopher S. Martin, Ph.D.
  Department of Psychiatry
  Western Psychiatric Institute and Clinic
  University of Pittsburgh School of Medicine
  3811 O’Hara Street
  Pittsburgh, PA 15213
Ann S. Masten, Ph.D.
  Director, Institute of Child Development
  University of Minnesota
  51 East River Road
  Minneapolis, MN 55455–0345
Matthew K. McGue, Ph.D.
  Psychology Department
  N218 Elliott Hall
  University of Minnesota
  75 East River Road
  Minneapolis, MN 55455–0344
Frank A. Middleton, Ph.D.
  Assistant Professor
  Departments of Psychiatry and Neuroscience and Physiology
  Director, Center for Neuropsychiatric Genetics
  Director, Microarray Core Facility
  State University of New York Upstate Medical University
  750 East Adams Street
  Syracuse, NY 13210
Stacia A. Murphy
  President
  National Council on Alcoholism and Drug Dependence, Inc.
  20 Exchange Place, Suite 2902
  New York, NY 10005
Daniel Pine, M.D.
  Chief, Section on Development and Affective Neuroscience and Chief of Child and Adolescent Research
  Mood and Anxiety Disorders Program
  National Institute of Mental Health
  Building 15K, Room 110
  Bethesda, MD 20892
John E. Schulenberg, Ph.D.
  University of Michigan Institute for Social Research
  P.O. Box 1248
  Room 2138
  Ann Arbor, MI 48106–1248
Kenneth J. Sher, Ph.D.
  Professor
  Department of Psychological Studies
  University of Missouri
  200 South Seventh Street
  Columbia, MO 65211–0001
Linda Spear, Ph.D.
  Distinguished Professor of Psychology
  Chair of Department of Psychology
  Binghamton University
  State University of New York
  P.O. Box 6000
  Binghamton, NY 13902–6000
H. Scott Swartzwelder, Ph.D.
  Professor of Psychiatry and Behavioral Sciences
  Senior VA Research Career Scientist
  Building 16, Room 24
  VA Medical Center
  508 Fulton Street
  Durham, NC 27705
Susan F. Tapert, Ph.D.
  Department of Psychiatry
  University of California, San Diego
  VA San Diego Healthcare System
  3350 La Jolla Village Drive, 116B
  San Diego, CA 92161
Michael Windle, Ph.D.
  Professor of Psychology
  University of Alabama-Birmingham
  Center for the Advancement of Youth Health
  912 Building, 1530 3rd Avenue, South
  Birmingham, AL 35294–1200
Robert A. Zucker, Ph.D.
  Professor of Psychiatry and Psychology
  Addiction Research Center
  University of Michigan
  400 East Eisenhower Pkwy., Suite 2A
  Ann Arbor, MI 48108–3318

## NIAAA CONTRIBUTORS

Charlotte Armstrong
  Office of Research Translation and Communications
Judith Arroyo, Ph.D.
  Division of Epidemiology and Prevention Research
Gregory Bloss, M.A.
  Division of Epidemiology and Prevention Research
John Bowersox
  Office of Research Translation and Communications
Gayle Boyd, Ph.D.
  (now at Center for Scientific Review, NIH)
  Division of Epidemiology and Prevention Research
Vivian B. Faden, Ph.D.
  Division of Epidemiology and Prevention Research
Thomas Gentry, Ph.D.
  Division of Metabolism and Health Effects
David Goldman, M.D.
  Division of Intramural Clinical and Biological Research
Mark Goldman, Ph.D.
  Office of the Director
Roger Hartman, M.L.S.
  Office of Research Translation and Communications
Ralph Hingson, Sc.D., M.P.H.
  Division of Epidemiology and Prevention Research
Cherry Lowman, Ph.D.
  Division of Treatment and Recovery Research
Howard Moss, M.D.
  Office of the Director
Patricia Powell, Ph.D.
  Office of Scientific Affairs
Gregory Roa
  Office of Research Translation and Communications
Kathy Salaita, Ph.D.
  (now at Center for Scientific Review, NIH)
  Division of Epidemiology and Prevention Research
Roger Sorensen, Ph.D.
  Division of Neuroscience and Behavior
Ellen Witt, Ph.D.
  Division of Neuroscience and Behavior

